# Thermal Condensation of Dehydrogenation Polymer (DHP) with Xylose

**DOI:** 10.3390/polym16223139

**Published:** 2024-11-11

**Authors:** Peng Wang, Jiaju Xie, Wenyao Peng, Junxian Xie, Junjian An, Guangyan Zhang, Junjun Chen

**Affiliations:** 1 Hubei Provincial Key Laboratory of Green Materials for Light Industry, Hubei University of Technology, Wuhan 430068, China; 2 School of Materials and Chemical Engineering, Hubei University of Technology, Wuhan 430068, China

**Keywords:** dehydrogenation polymer, xylose, thermal condensation mechanism, adhesives

## Abstract

Conventional adhesives used in wood-based panels typically contain volatile organic compounds, including formaldehyde, which can potentially lower indoor air quality and damage human health. Lignin, a natural adhesive present in wood, offers significant advantages over other materials due to its ready availability, renewable nature, rich aromatic rings, and aliphatic and aromatic hydroxyl groups, as well as quinone groups. However, when modified as an adhesive for wood-based panels, lignin suffers from poor water resistance and formaldehyde release. Dehydrogenation polymer (DHP), as a lignin model compound, possesses a structure similar to lignin and excellent water resistance, making it a potential substitute for lignin as a formaldehyde-free adhesive. A DHP-xylose complex was obtained from a condensation reaction between DHP and xylose in hemicellulose in a simulated hot-pressing environment. The feasibility of DHP bonding with hemicellulose components was verified using FT-IR and NMR spectroscopic methods. In addition, the structure of the adduct and condensation process were also studied. DHP and xylose underwent condensation under simulated hot-pressing conditions. Xylose and DHP may be linked by C-C bonds. The thermal condensation of DHP with xylose was investigated. This may contribute to a better understanding of the adhesive bonding process for xylose during hot-pressing and offer support for practical applications.

## 1. Introduction

In the contemporary world, there is a continuously increasing demand in the construction and furniture industries for high-performance, environmentally friendly materials. As an indispensable part of this field, wood-based panels occupy an important position in several areas. Traditional wood-based panels are fabricated from wood or other non-wood plant materials, which are subjected to mechanical processing to segregate them into distinct unit materials, which are subsequently bonded with adhesives to produce boards or molded products. The core of wood-based panel production lies in using adhesives to firmly bond wood particles or fibers together, forming a sturdy panel structure. Adhesives play a crucial role in the production of wood-based panels and directly influence the performance, quality, and lifespan of the resulting boards. Traditional adhesives used in the production of wood-based panels often include urea-formaldehyde, phenol-formaldehyde, melamine, and their modified derivatives [1]. Although these adhesives have strong bonding strength and water resistance, they also release harmful volatile organic compounds (VOCs) such as formaldehyde and phenol, posing potential risks to indoor air quality and human health [2].

To mitigate or eliminate formaldehyde pollution in wood-based panels, more environmentally friendly biomass adhesives have become a focus. Among numerous biomass adhesives, lignin-based adhesives have been extensively studied. They are typically derived from industrial lignin, a by-product of the paper industry, which has the characteristics of being easy to obtain and low-cost compared with traditional adhesives. Lignin-based adhesives are usually prepared by blending modified industrial lignin with “three-aldehyde” resins, which can reduce the release of toxic substances [3,4,5,6,7,8,9,10,11,12]. However, the issue of formaldehyde emissions has not been completely solved. After enzymatic modification, industrial lignin promotes the formation of a large number of radicals during the production of wood-based panels [13,14]. These radicals promote the cross-linking of industrial lignin through mutual coupling, thereby improving its bonding performance while completely eliminating the problem of formaldehyde emission. Wood-based panels with high strength have been obtained by treating industrial lignin with laccase [13,14,15]. However, the enzyme modification of lignin is only effective in an aqueous solution. Although the modified wood boards have good dry strength, their water resistance is relatively poor [13,14]. Therefore, the potential for using enzymatically modified industrial lignin to prepare formaldehyde-free adhesives is subject to significant limitations. The enzyme-catalyzed transformation of phenolic substances or lignin precursors can generate a dehydrogenation polymer (DHP) with a structure resembling that of lignin. This material may serve as an adhesive [16,17,18]. The polymer can couple with free phenolic groups at the surface of thermomechanical pulp (TMP) fibers under catalytic oxidation promoted by enzymes to significantly improve the wet strength of paper. Laccase and peroxidase have been utilized to prepare DHP on the fiber surface. A significant enhancement in water resistance for paper and paperboard was observed [17,18,19,20,21,22,23,24]. In summary, the use of DHP to function as an adhesive in wood panels has been investigated.

In natural wood, lignin and cellulose fibers are primarily cross-linked through high-molecular-weight lignin and hemicellulose. The lignin and hemicellulose can form lignin–carbohydrate complexes (LCCs) through covalent bonding. These two interactions account for the excellent physical and mechanical properties of wood [25]. Therefore, two conditions need to be met for DHP to function as an effective adhesive. Firstly, it must efficiently self-condense during hot-pressing to form high-molecular-weight polymers that cross-link wood fibers. Secondly, high-molecular-weight DHP must condense with hemicellulose to form covalently bonded structures similar to LCCs. This allows DHP to tightly bind plant cell walls together through both physical adhesion and chemical bonding. This ensures good adhesive performance in wood-based panels. The fact that DHP can efficiently undergo self-condensation under hot-pressing conditions to form high polymers [26] has previously been demonstrated. This suggests that DHP has potential to be used as an adhesive.

Consequently, whether or not high-molecular-weight DHP can undergo condensation with hemicellulose under hot-pressing conditions to form covalently bonded structures similar to those of LCCs has been investigated. Specifically, the reaction of DHP with xylose in hemicellulose under simulated hot-pressing conditions has been examined. Moreover, the structure and thermal condensation process for DHPs with hemicellulose have been investigated using FT-IR and NMR spectroscopic methods. Groundwork for the development of high-performance wood-based panel adhesives that are environmentally friendly and non-polluting has been established.

## 2. Materials and Methods

### 2.1. Materials

Acetic Acid, Toluene, and Dimethyl Sulfoxide (DMSO), all from Analytical Reagent, were purchased from Sinopharm Chemical Reagent Co., Ltd. (Shanghai, China) and xylose was purchased from Shanghai Aladdin Biochemical Technology Co., Ltd. (Shanghai, China). The synthesis of DHP is referenced in [27,28].

### 2.2. Preparation of DHP-Xylose Complex

Xylose (200 mg), DHP (120 mg), and 200 μL of acetic acid solution (10% *v/v*) were added to a 2 mL stainless steel sealed vessel and heated in an oil bath at 140 °C for 25 min. After the reaction, we performed repeated washes and centrifugation with distilled water and removed the supernatant to ensure that the xylose was completely removed until the wash solution was neutral. The resulting insoluble substance was the final product, which was then dried to obtain the DHP-xylose complex. These experimental conditions were based on previous research conducted by our group [26].

### 2.3. Structural Characterization of DHP-Xylose Complex

The DHP-xylose complex was ball-milled for about 22 h. The ball-milled product was washed with toluene, centrifuged at a low temperature, and dried to obtain the powdered product. A 2 mg sample was mixed with 250 mg KBr (ground in an agate mortar) and pressed into a pellet, and the infrared spectra of the DHP self-condensation product (DHP SC) and the DHP-xylose complex (DHP-Xylose) were recorded using a Fourier transform infrared spectrometer (model: NICOLET 6700, Waltham, MA, USA). An amount of 100 mg of DHP-xylose was dissolved in 1.2 mL of DMSO-d6 in a 5 mm NMR tube and detected using a 500 MHz AVANCE III Nuclear Magnetic Resonance Spectrometer (Bruker, Berlin, Germany) with 9000 scans. Then, 80 mg of DHP-xylose was dissolved in 1 mL of DMSO-d6 in a 5 mm NMR tube and analyzed using a 500 MHz AVANCE III Nuclear Magnetic Resonance Spectrometer (Bruker, Berlin, Germany) with 32 scans. Resolution: ^1^H: 0.45 Hz, ^13^C: 0.2 Hz; sensitivity: ^1^H ≥ 730:1 (0.1% EB), ^13^C ≥ 250:1 (ASTM).

## 3. Results and Discussions

### 3.1. FT-IR Analysis of DHP-Xylose Complex

**Figure 1:**
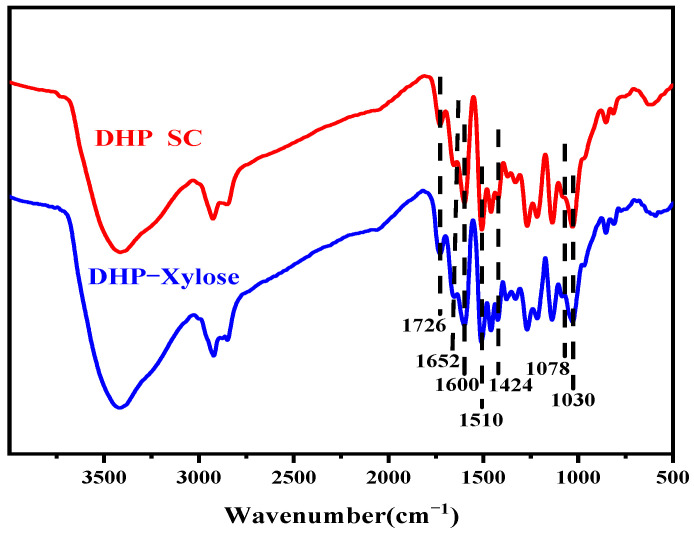
FT-IR spectra of DHP self-condensation and DHP-xylose complex.

The infrared spectra of DHP self-condensation and the DHP-xylose complex are shown in Figure 1. The absorption peaks at 1078 cm^−1^, 1652 cm^−1^, and 1726 cm^−1^ in the spectrum correspond to the vibration of C-O in the ether bonds, the conjugated carbonyl group, and the non-conjugated carbonyl group, respectively. The signals of benzene rings were observed at 1600 cm^−1^, 1510 cm^−1^, and 1424 cm^−1^ in the spectrum of the DHP-xylose complex, indicating the presence of the benzene ring framework. Compared with self-condensed DHP, the absorption peaks at 1078 cm^−1^ and 1030 cm^−1^ in the spectrum of the DHP-xylose complex were intensified. The peak at 1078 cm^−1^ corresponded to the C-O vibration in the ether bond, while the peak at 1030 cm^−1^ was associated with the C=O stretching vibration of xylose [29], indicating the possible condensation of DHP with xylose. Further characterization required analysis via ^13^C-NMR.

### 3.2. ^13^C-NMR Analysis of DHP-Xylose Complex

**Figure 2:**
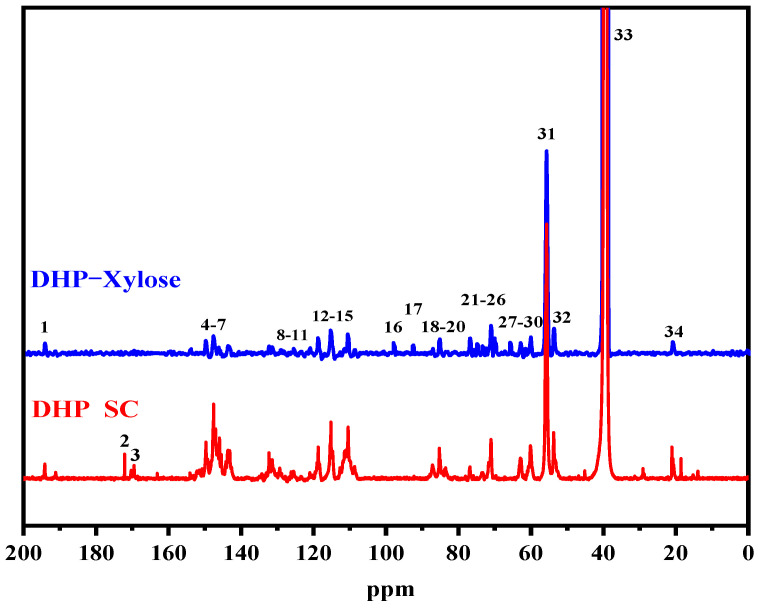
^13^C-NMR spectra of DHP self-condensation and DHP-xylose complex.

The ball-milled DHP-xylose complex was completely dissolved in DMSO. The ^13^C NMR spectra of self-condensed DHP and the DHP-xylose complex are shown in Figure 2, and the functional group assignments for both are provided in Table 1 [30]. As shown in Figure 2, the DHP-xylose complex exhibited several new absorption peaks compared to the self-condensed DHP. These include the C_1_ absorption peak of β-D-xylose at 98.3 ppm, the C_1_ absorption peak of α-D-xylose at 93 ppm, the C_3_ absorption peak of xylose at 75.3 ppm, the C_2_ absorption peak of β-D-xylose at 73.7 ppm, the C_2_ absorption peak of α-D-xylose at 72.9 ppm, the C_4_ absorption peak of xylose at 70.1 ppm, and the C_5_ absorption peak of xylose at 66.1 ppm [29]. Additionally, within the range of 60–64 ppm, a new absorption peak at 62.1 ppm (No. 29) appeared for the DHP-xylose complex, while the peaks at 63.2 ppm and 60.4 ppm remained largely unchanged. The peak at 62.1 ppm corresponded to the γ-C absorption peak of lignin, indicating a substantial increase in the lignin structure within the condensed DHP-xylose complex [31]. Moreover, a signal peak appeared around 85 ppm, corresponding to C_α_ and C_β_ [29]. These findings thoroughly demonstrate the thermal condensation of DHP with xylose under hot-pressing conditions.

### 3.3. Two-Dimensional-HSQC NMR Analysis of DHP-Xylose Complex

**Figure 3:**
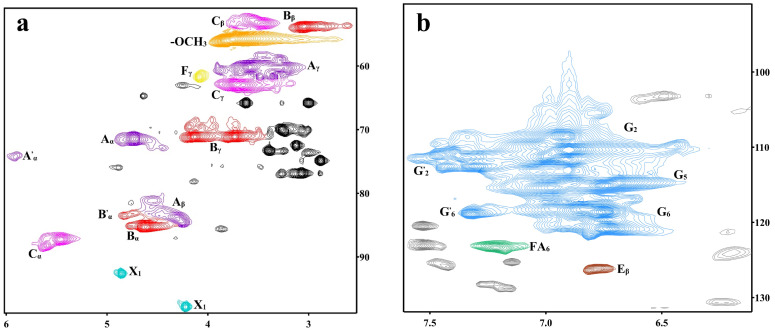
Two-dimensional HSQC NMR spectra of the DHP-xylose complex: (**a**) side-chain region and (**b**) aromatic ring region.

**Figure 4:**
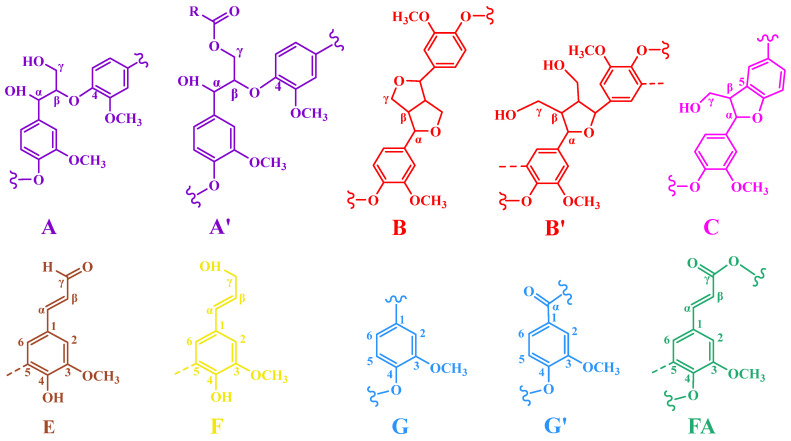
The main basic junction structures in the 2D-HSQC NMR of the DHP-xylose complex.

To further support the evidence of the condensation of DHP with xylose, 2D-HSQC NMR analysis was performed on the DHP-xylose complex. The side-chain region (δC/δH 50~100/2.2~6.2) and the aromatic ring region (δC/δH 90~135/5.9~7.8) of the 2D-HSQC NMR spectrum of the DHP-xylose complex are shown in Figure 3, and the functional group assignments of the main signals in the 2D-HSQC NMR spectrum are presented in Table 2 [30], while the primary linkage structures within the DHP-xylose complex are illustrated in Figure 4.

In the NMR spectra (Figure 3a,b, side-chain region and aromatic ring region), the side-chain structures (β-O-4, β-5, and β-β) and benzene ring structures (G_2_, G_5_, and G_6_) of DHP were observed. The signals at 92.62/4.86 ppm and 97.83/4.22 ppm (X_1_) [30,32,33] were attributed to the C_1_ signals of α-D-xylose and β-D-xylose, respectively. The signal at 74.26/5.91 ppm was identified as the β-O-4 and C_α_-H_α_ signal bond. DHP underwent thermal condensation with xylose under high-temperature, high-pressure, and acidic conditions, which was consistent with the previous analysis of FT-IR and ^13^C-NMR spectra.

**Figure 5:**
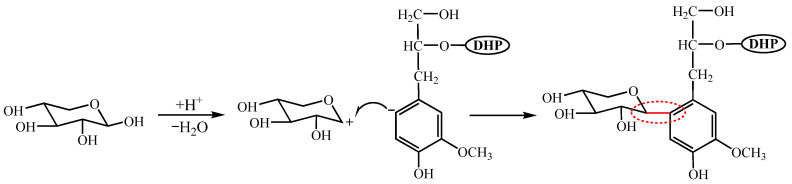
Thermal condensation of DHP with xylose.

In conclusion, the ^13^C NMR spectrum revealed the absorption signals of xylose C_1_, C_2_, C_3_, C_4_, and C_5_, confirming the presence of xylose. In the side-chain region of the 2D-HSQC NMR spectrum, along with the functional group assignment table, a chemical linkage between DHP and xylose was also found. It is speculated that the C_1_ of xylose and the C_6_ of DHP may be linked by C-C bonds [34,35]. The related formation process is illustrated in Figure 5.

## 4. Conclusions

DHP and xylose were used as raw materials to prepare DHP-xylose composites, catalyzed by a 10% volume fraction of acetic acid at 140 °C. The appearance of signals corresponding to xylose in the infrared spectrum of the DHP-xylose complex confirmed that DHP had undergone condensation with xylose under simulated hot-pressing conditions. Additionally, xylose-related signals were also observed in the NMR spectrum, indicating that DHP and xylose may be linked by C-C bonds, forming covalently bonded structures similar to LCCs. This represents a new opportunity to replace traditional formaldehyde adhesives, thereby promoting development in the formaldehyde-free adhesive field.

## Figures and Tables

**Table 1:** Functional group assignments of DHP self-condensation and DHP-xylose complex.

Signal	Chemical Shifts (δ, ppm)		Assignments
	DHP SC	DHP-Xylose	
1	194.1	194.5	γ-CHO in cinnamaldehyde
2	172.1	—	C_γ_ in Ferulic acid
3	169.5	—	-COO- in Ferulic acid ester
4	150.6	150.1	C_3_/C_4_ in guaiacyl
5	147.5	148.1	C_3_/C_5_ in guaiacyl, etherified
6	145.9	146.6	C_4_/C_4_′ in 5-5′, etherified
7	143.6	144	C_4_ in 5-5
8	132.2	132.7	C_1_ in β-O-4 guaiacyl, non-etherified
9	131.3	131.9	C_1_/C_1_′ in β-5
10	129.3	129.4	C_α_ in cinnamaldehyde
11	128.7	128.6	C_β_ in cinnamaldehyde
12	119.5	119.3	C_6_ in guaiacyl
13	118.7	118.6	C_5_ in guaiacyl
14	115.3	115.8	C_5_ in guaiacyl
15	110.4	110.9	C_2_ in guaiacyl
16	—	98.3	C_1_ in β-D-Xylose
17	—	93	C_1_ in α-D-Xylose
18	87.1	87.5	C_α_ in β-5
19	85	85.5	C_α_(β-β), C_β_(β-O-4)
20	83.8	83.5	C_β_ in β-O-4
21	76.9	77.3	C_α_ in β-O-4
22	—	75.3	C_3_ in Xylose
23	—	73.7	C_2_ in β-D-Xylose
24	—	72.9	C_2_ in α-D-Xylose
25	71	71.5	C_γ_ in β-β
26	—	70.1	C_4_ in Xylose
27	—	66.1	C_5_ in Xylose
28	62.9	63.2	C_γ_ in β-5
29	—	62.1	C_γ_ in cinnamylalcohol
30	60.1	60.4	C_γ_ in β-O-4
31	55.6	56.2	-OCH_3_
32	53.7	54	C_β_ in β-5
33	39.4	40	DMSO
34	21	21.3	-CH_3_ in acetyl group

**Table 2:** Two-dimensional HSQC NMR functional group assignments of the DHP-xylose complex.

Label	Chemical Shift	Assignments
	δ_C_/δ_H_ (ppm)	
C_β_	53.29/3.46	C_β_-H_β_ in phenylcoumaran (C)
B_β_	53.82/3.04	C_β_-H_β_ in β-β (resinol) (B)
OCH_3_	55.82/3.76	C-H in methoxyls
A_γ_	60.19/3.59	C_γ_-H_γ_ in β-O-4 substructures (A)
F_γ_	61.73/4.08	C_γ_-H_γ_ in cinnamyl alcohol end-groups (F)
C_γ_	62.96/3.71	C_γ_-H_γ_ in phenylcoumaran (C)
Bγ	71.10/3.73	C_γ_-H_γ_ in β-β resinol (B)
	71.13/4.13	
A_α_	71.41/4.73	C_α_-H_α_ in β-O-4 unit (A)
A′_α_	74.26/5.91	β-O-4, C_α_-H_α_ in guaiacyl units (G)
A_β_	83.99/4.29	C_β_-H_β_ in β-O-4 substructures (A)
	81.18/4.56	
	83.50/4.48	
B_α_	85.28/4.62	C_α_-H_α_ in β-β resinol (B)
B′_α_	83.40/4.83	C_α_-H_α_ in β-β (B′, tetrahydrofuran)
C_α_	87.22/5.46	C_α_-H_α_ in phenylcoumaran (C)
X_1_	92.62/4.86	C_1_-H_1_ in α-D-Xylose
	97.83/4.22	C_1_-H_1_ in β-D-Xylose
G_2_	108.55/6.92	C_2_-H_2_ in guaiacyl units (G)
	110.38/6.90	
G′_2_	110.71/7.39	α C_2_-H_2_ in G-type structural units with oxidized sites
	112.58/7.31	
G_5_	115.13/6.74	C_5_-H_5_ in guaiacyl units (G)
	115.37/6.98	
G_6_	118.61/6.77	C_6_-H_6_ in guaiacyl units (G)
	120.84/6.74	
G′_6_	118.81/7.32	α C_6_-H_6_ in G-type structural units with oxidized sites
FA_6_	123.30/7.21	C_6_-H_6_ in ferulate (p-FA)
E_β_	126.17/6.78	C_β_-H_β_ in cinnamyl aldehyde end-groups (E)

## Data Availability

Data are contained within the article.
